# Genetic parameters and signatures of selection in two divergent laying hen lines selected for feather pecking behaviour

**DOI:** 10.1186/s12711-015-0154-0

**Published:** 2015-09-30

**Authors:** Vanessa Grams, Robin Wellmann, Siegfried Preuß, Michael A. Grashorn, Jörgen B. Kjaer, Werner Bessei, Jörn Bennewitz

**Affiliations:** Institute of Animal Science, University of Hohenheim, 70593 Stuttgart, Germany; Institute for Animal Welfare and Animal Husbandry, Friedrich-Loeffler-Institut, Doernbergstrasse 25-27, 29223 Celle, Germany

## Abstract

**Background:**

Feather pecking (FP) in laying hens is a well-known and multi-factorial behaviour with a genetic background. In a selection experiment, two lines were developed for 11 generations for high (HFP) and low (LFP) feather pecking, respectively. Starting with the second generation of selection, there was a constant difference in mean number of FP bouts between both lines. We used the data from this experiment to perform a quantitative genetic analysis and to map selection signatures.

**Methods:**

Pedigree and phenotypic data were available for the last six generations of both lines. Univariate quantitative genetic analyses were conducted using mixed linear and generalized mixed linear models assuming a Poisson distribution. Selection signatures were mapped using 33,228 single nucleotide polymorphisms (SNPs) genotyped on 41 HFP and 34 LFP individuals of generation 11. For each SNP, we estimated Wright’s fixation index (F_ST_). We tested the null hypothesis that F_ST_ is driven purely by genetic drift against the alternative hypothesis that it is driven by genetic drift and selection.

**Results:**

The mixed linear model failed to analyze the LFP data because of the large number of 0s in the observation vector. The Poisson model fitted the data well and revealed a small but continuous genetic trend in both lines. Most of the 17 genome-wide significant SNPs were located on chromosomes 3 and 4. Thirteen clusters with at least two significant SNPs within an interval of 3 Mb maximum were identified. Two clusters were mapped on chromosomes 3, 4, 8 and 19. Of the 17 genome-wide significant SNPs, 12 were located within the identified clusters. This indicates a non-random distribution of significant SNPs and points to the presence of selection sweeps.

**Conclusions:**

Data on FP should be analysed using generalised linear mixed models assuming a Poisson distribution, especially if the number of FP bouts is small and the distribution is heavily peaked at 0. The F_ST_-based approach was suitable to map selection signatures that need to be confirmed by linkage or association mapping.

**Electronic supplementary material:**

The online version of this article (doi:10.1186/s12711-015-0154-0) contains supplementary material, which is available to authorized users.

## Background

Feather pecking (FP) in laying hens is a well-known, but yet unsolved problem. This abnormal behaviour is characterized by non-aggressive pecks directed towards the plumage of other hens [1]. It causes economic losses due to increases in feeding costs when large parts of the body are denuded and in mortality rates when FP leads to cannibalism. In addition to a number of environmental conditions, physiological, nutritional as well as genetic and epigenetic factors are known to influence FP (see [2–6]). Quantitative genetic analyses have reported heritability estimates in the range of 0.1 to 0.4, depending on the trait definition, design of the study, age of the hens, statistical model applied, and data collection period [1, 7–9]. FP shows a complex genetic relationship with other traits such as feather eating and number of laid eggs [10, 11], traits related to aggressiveness and fear response [12–14], and general activity and explorative behaviour [15].

Kjaer et al. [1] carried out a selection experiment to develop a high feather pecking line (HFP) and a low feather pecking line (LFP), starting from a common base population. After two rounds of selection, FP was significantly more pronounced in the HFP line than the LFP line. Su et al. [2] used the data from the first five generations of these lines to estimate variance components and heritabilities. Heritability ranged from 0.11 to 0.17. Five additional rounds of selection were then conducted. Our aim was to perform a quantitative genetic analysis of FP on animals from these additional rounds of selection in order to discuss the data obtained with those reported by Su et al. [2], and to determine the best approach for analyzing such data.

As indicated by Wysocki et al. [5], performing a genome-wide study to map QTL (quantitative trait loci) or genes that underlie genetic variation of FP would help to better understand this abnormal behaviour and its complex relationships with other traits. QTL linkage and association mapping rely on genotypes and phenotypes that are preferably collected from a large-scale study. However, since FP is not recorded in routine breeding programs, such large-scale designs cannot rely on existing datasets and need to be established, which is a time-consuming and costly effort, because observing and recording FP is labour intensive.

Based on Qanbari and Simianer [16], selection signatures are defined as regions of the genome that harbour functionally important sequence variants and have changed under selection. It is well known that strong selection leads to reduced nucleotide diversity around the loci under selection. Not only is the diversity of the target loci reduced, but also that of loci in high linkage disequilibrium (LD) with the target loci. This is known as genetic hitch-hiking [17] and results in selection signatures in the genome. Mapping selection signatures has been a matter of intense research during the last years; see [16, 18] and references in these two papers. A genome scan to map selection signatures requires a dense genetic map in order to exploit LD. In chicken, a 60 K SNP (single nucleotide polymorphism) Illumina iSelect chip was developed by the USDA Chicken GWMAS Consortium. Kranis et al. [19] reported the development of a 600 K Affymetrix HD genotyping chicken array. A large range of methods is available for the detection of selection signatures, which can be classified according to whether intra- or inter-population information is used. To analyze inter-population information, Wright’s fixation index, F_ST_, is widely used, for which several estimators are described [20, 21].

Given the availability of dense SNP chicken arrays, an alternative to using linkage or association mapping to detect QTL for FP, is to search for selection signatures using data from the last generation of the HFP and LFP lines. This approach could lead to the identification of chromosomal regions that contain genes having responded to divergent selection, and hence, contribute to the genetic variation of FP. Therefore, our second aim was to conduct a genome scan to map selection signatures based on data from the last generation of the selection experiment described above by applying the F_ST_ statistic.

## Methods

### Animals, data collection and selection

Chickens of a White Leghorn layer line were divergently selected for high and low FP for 11 generations. The selection started in the Danish Institute of Animal Sciences, Foulum, Denmark, for the first six generations (0–5) [1]. Thereafter, five rounds of selection took place at the Institute of Animal Science, University of Hohenheim, Germany. The common base population of both lines was established in 1995 and derived from a foundation stock, which was created in 1970 as a control population in the Scandinavian selection and cross-breeding experiment of Liljedahl et al. [22], see also Kjaer et al. [1] and Su et al. [2]. In the base population (generation 0), FP was recorded on 123 hens at the age of 67 weeks. This information was used to estimate breeding values and, then, 30 females and 10 males with the highest and lowest estimated breeding value for FP were selected as the founder animals of the HFP and LFP lines, respectively.

This selection procedure was repeated in the subsequent generations. Up to generation 5, at about 30 weeks of age, groups of 20 hens (10 HFP and 10 LFP) per pen were transferred into observation pens (size 2 m × 4 m). The observation period started 7 to 12 days after the hens were transferred to the observation pens. Feather pecking behaviour was recorded by video camera during 3 h and the number of FP bouts was counted for each hen. An FP bout was defined as a series of continuous pecks directed to the same part of the body of a recipient hen. At each generation, 10 males and 30 females per line were selected based on their breeding value for the number of FP bouts. For a detailed description of the experiment and the results of the statistical analysis, see Kjaer et al. [1] and Su et al. [2].

Behaviour testing and the selection procedure from generation 6 to 11 were carried out at the experimental farm of the University of Hohenheim. At about 30 (25 to 37) weeks of age, groups of 40 hens (20 HFP and 20 LFP) per pen were transferred into floor pens measuring 16 m^2^. For individual identification, a plastic tag was attached to the back of each bird. The observation period started 1 week after the birds were transferred to the floor pens and the number of FP bouts (defined as above) was counted for each hen. Each pen was observed by each observer (one observer per one pen at a time) during sessions of 20 min over three consecutive days. Each hen was observed during a total of 3 h. Selection was based on the number of FP bouts. At each generation and for each line, 60 females and about 10 males were selected based on their estimated breeding value that was calculated using an animal model. In this study, observation records and pedigree data were available only from generation 6 onwards. In total, 1526 hens were phenotyped for FP behaviour from generation 6 to 11. The research project was approved by the University of Hohenheim Committee of Animal Care and the Provincial Government of Baden-Wuerttemberg, under the authorisation number HOH 35/15PG.

### Estimation of variance components

Statistical analyses of the data recorded during the last six generations were performed using an animal model and the ASREML software package [23]. Two different models were used, i.e. a generalized linear mixed model and a linear mixed model. In both models, HFP and LFP lines were analyzed separately. The vector containing the linear predictors of the observations (**η** = {η_i_}) was:

1 $$ \varvec{\upeta} = {\mathbf 1}\mu + {\mathbf Z_{\mathbf {gen}} \mathbf {gen}} + {\mathbf Z_{\mathbf a} \mathbf a}, $$

where *µ* is the intercept, **gen** is a vector with random generation effects, **a** is a vector with the random additive-genetic effects, and **Z**_**gen**_ and **Z**_**a**_ are known design matrices. Covariance structures of random effects were var(**gen**) = **I** × σ_gen_^2^ and var(**a**) = **A** × σ_a_^2^, where *σ*_*gen*_^2^ and *σ*_*a*_^2^ are generation variance and additive genetic variance, respectively, and **A** and **I** are the numerator relationship and identity matrices, respectively. An observation was equal to the number of FP bouts recorded over the entire observation period and stored in vector **y**. Expectations of the observations were as follows:

$$ \varvec{\uplambda} = {\mathrm E}\left( {\mathbf {y|gen,a}} \right) = {\mathrm g^{ - 1}} \left( \varvec {\upeta} \right),$$

where $\varvec{\uplambda}$ = {*λ*_*i*_} is a vector containing the Poisson parameters of the observations and g is the link function, in this case log link.

In the Poisson model, the residual variance is not an explicit part of the model. If estimating the heritability is of interest, the residual variance has to be modelled entirely on the link scale. Formulas to do this are given in Foulley et al. [24] and Bennewitz et al. [10].

**Fig. 1:**
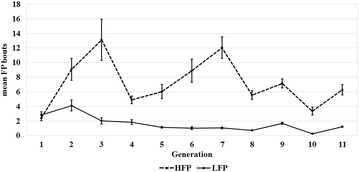
Phenotypic trend over 11 generations. The *interrupted* and *continuous lines* show the average number of feather pecking bouts per hen during the observation period of 180 min with respective standard errors for the high and low feather pecking line

For the analysis of the data using the linear mixed model, the observations were Box-Cox transformed as follows:

$$y_{ti} = \frac{{\left( {y_{i}^{ - 0.2} - 1} \right)}}{ - 0.2},$$

where *y*_*i*_ is the number of FP bouts for each hen *i* summed up over the entire observation period and *y*_*ti*_ is the transformed observation. The power parameter was −0.2, which was found to give the best fit of the model applied by Su et al. [2] using data from the first six generations of the same selection experiment. The following mixed model was used:

2 $${\mathbf y_{t}} = {\mathbf 1}\mu + {\mathbf Z_{\mathbf {gen}} \mathbf {gen}} + {\mathbf Z_{\mathbf {a}} \mathbf {a}} + e,$$

where **y**_**t**_ is the vector of transformed observations, *e* denotes the random residual and the remaining terms are as defined in model (1). HFP and LFP lines were analyzed separately, because pedigree information was not available up to the common base population and trait means differed constantly between the two lines across generations (Fig. 1). A generation effect was included to capture the large fluctuations in the means for each generation that were observed from generation 6 onwards. This effect also captures at least part of the putative genetic progress across generations, a point that will be discussed later. In this model, heritability was estimated using standard procedures.

### Genotyping

Genotyping was performed on 41 HFP and 34 LFP hens from generation 11 using the Illumina 60 K chicken Infinium iSelect chip. A total of 57,636 SNPs were detected and after control checks, 33,228 remained for the statistical analyses described below. SNPs that were located on one of the sex chromosomes W or Z or on linkage groups LGE22C19W28_E50C23 or LGE64, respectively, and SNPs that were not allocated to a specific chromosome or linkage group were excluded. In addition, monomorphic SNPs [minor allelic frequency (MAF) = 0.0] and SNPs with a call frequency less than 0.95 were filtered out. The remaining SNPs were checked for correct clustering by Illumina’s GenomeStudio software. For this purpose, SNPs were sorted consecutively by using different metrics (heterozygote excess, cluster separation, parent-parent-child errors) and those that showed extreme values were checked visually for correct clustering and, where appropriate, were manually re-clustered. SNPs for which a manual re-clustering was not possible were excluded from analyses.

### Estimation of F_ST_ index and mapping of selection signatures

To identify regions under selection, we used the population differentiation index F_ST_. In general, F_ST_ provides a measure to quantify levels of differentiation between subpopulations [20, 25]. A small F_ST_ (e.g. <0.05) indicates that allele frequencies in both subpopulations are similar, whereas an F_ST_ greater than 0.05 indicates that allele frequencies are different. We used the F_ST_ computation of Weir and Cockerham (Equation 8 in [25]), which is for a single SNP:

3 $${\text{F}}_{\text{ST}} = \frac{{\sigma_{p}^{2} }}{{\bar{p}(1 - \bar{p})}},$$

where $\bar{p}$ is the mean allele frequency for the two lines and *σ*_*p*_^2^ is the variance of the allele frequency across the two lines. *σ*_*p*_^2^ is estimated as $\sigma_{p}^{2} = \left( {\overline{{p^{2} }} } \right) - \left( {\bar{p}^{2} } \right)$, where $\overline{{p^{2} }}$ is the mean of the squared allele frequencies in the two lines.

Single F_ST_ values can vary greatly. In addition, selection sweeps will affect the F_ST_ of consecutive SNPs due to the LD between them. Therefore, we also calculated F_ST_ for sliding windows that each consisted of 25 SNPs and moved in steps of one SNP forward. The computation was done using the following formula, which is a multi-marker extension of (3):

$${\text{F}}_{\text{ST}} = \frac{\sum \nolimits_{i}{\left[ {\left( {\overline{{p_{i}^{2} }} } \right) - \left( {\bar{p}_{i}^{2} } \right)} \right]}}{{\mathop \sum \nolimits_{i} \overline{{p_{i} }} \left( {1 - \overline{{p_{i} }} } \right)}},$$

where index *i* denotes the *i*th SNP in the sliding window.

In our experiment, the differences in allele frequencies between the two lines could be driven by genetic drift and selection. To unravel these two processes, a statistical test was developed, which is based on the assumption, that genetic drift affects the whole genome, while selection affects only SNPs that are in LD with causal genes. F_ST_ values were used as test statistics. For each SNP, we tested the null hypothesis that F_ST_ was driven purely by genetic drift against the alternative hypothesis that it was driven by genetic drift and selection. To derive a null distribution of the test statistic, we simulated the effect of genetic drift stochastically. We were able to do this, because, as described above, the breeding history of each line starting from the common base population was known. In the first five rounds of selection, 10 males and 30 females were selected in each line [2], which resulted in an effective population size (Ne) of 30. In the next five rounds of selection, the number of females was increased to about 60, resulting in a Ne of approximately 35. Since genetic drift is largest for intermediate allele frequencies, the allele frequency in the base population was assumed to be equal to 0.5. Two populations with one SNP were simulated from the common base population and were bred for 11 generations independently by assuming a Ne of 30 for the first five rounds of selection and 35 for the next five rounds of selection. At generation 11, F_ST_ for these two populations at the SNP was computed using formula (2). This was repeated 100,000 times and resulted in a distribution of F_ST_ values under the null hypothesis of no selection.

The error probability for each real SNP (*p*_*nominal*_) was computed as the proportion of simulated SNPs that had a greater F_ST_ than the real SNP under consideration. To correct for multiple testing, we applied the Bonferroni correction as *p*_*genomewide*_ = 1 − (1 − *p*_*nominal*_)^#*SNP*^, where the number of SNPs was equal to 33,228. The genome-wide significance level was set at *p*_*genomewide*_ ≤0.05. Because the Bonferroni correction is very conservative due to the assumption of independence of tests (which is not the case in our study due to the LD structure of consecutive SNPs and due to selection), we considered two additional levels of significance, i.e. *p*_*nominal*_ ≤5 × 10^−5^, and *p*_*nominal*_ ≤5 × 10^−4^. In order to estimate the number of false positives among the significant SNPs, we calculated false discovery rates (FDR).

### Clustering

As denoted above, it is likely that selection led to increased F_ST_ indexes for a series of consecutive SNPs. Therefore, we identified clusters of SNPs, which provided stronger evidence of selection sweeps, compared to F_ST_ indexes for single SNPs. A cluster contained a minimum of two significant SNPs (*p*_*nominal*_ ≤5 × 10^−5^) with a maximum distance of 3 Mb between them.

## Results

The phenotypic trend for feather pecking during the 11 generations of selection is shown in Fig. 1. From the first round of selection onwards, lines HFP and LFP differed in mean numbers of FP bouts. Selection response on the phenotypic scale was greatest during the first two rounds of selection, then, the mean number of FP bouts decreased sharply from generation 3 to 4 for the HFP line. The explanation is that HFP males were killed by accident in generation 3 and had to be replaced by males from a control line to produce the next generation. After generation 4, variability in pecking behaviour is most likely caused by environmental effects. For line LFP, the level of pecking behaviour was constantly low during the 11 generations of selection and showed only a small and almost undetectable decrease in the mean number of FP bouts over generations.

Estimated variance components are in Table 1. With the Poisson model (model 1), additive genetic variance for line HFP is almost twice as large as that for line LFP. However, even in line LFP, this variance is significantly different from zero since it has a small standard error. Generation variance is small compared to additive genetic variance and its value is similar in both lines. In contrast, the generation variance estimated with the linear mixed model (model 2) is substantially larger, compared to the additive genetic variance. The heritability of FP for the HFP line was equal to 0.15 and was at the lower bound of the range of values reported in the literature. For line LFP, the additive genetic variance and hence the heritability were close to zero.

**Table 1:** Estimated additive genetic variance (*σ*_*a*_^2^), generation variance (*σ*_*gen*_^2^), residual variance (*σ*_*e*_^2^), heritability (h^2^) and standard error (in parenthesis) for trait feather pecking bouts using a Poisson model and a linear model

Model	Line	*σ*_*a*_^2^	*σ*_*gen*_^2^	*σ*_*e*_^2^	h^2^
Poisson	HFP	2.760 (0.24)	0.27 (0.23)	–	–
	LFP	1.430 (0.15)	0.35 (0.24)	–	–
Linear	HFP	0.090 (0.04)	0.08 (0.05)	0.55 (0.04)	0.15 (0.07)
	LFP	0.001 (0.01)	0.03 (0.02)	0.26 (0.02)	0.01 (0.03)

**Fig. 2:**
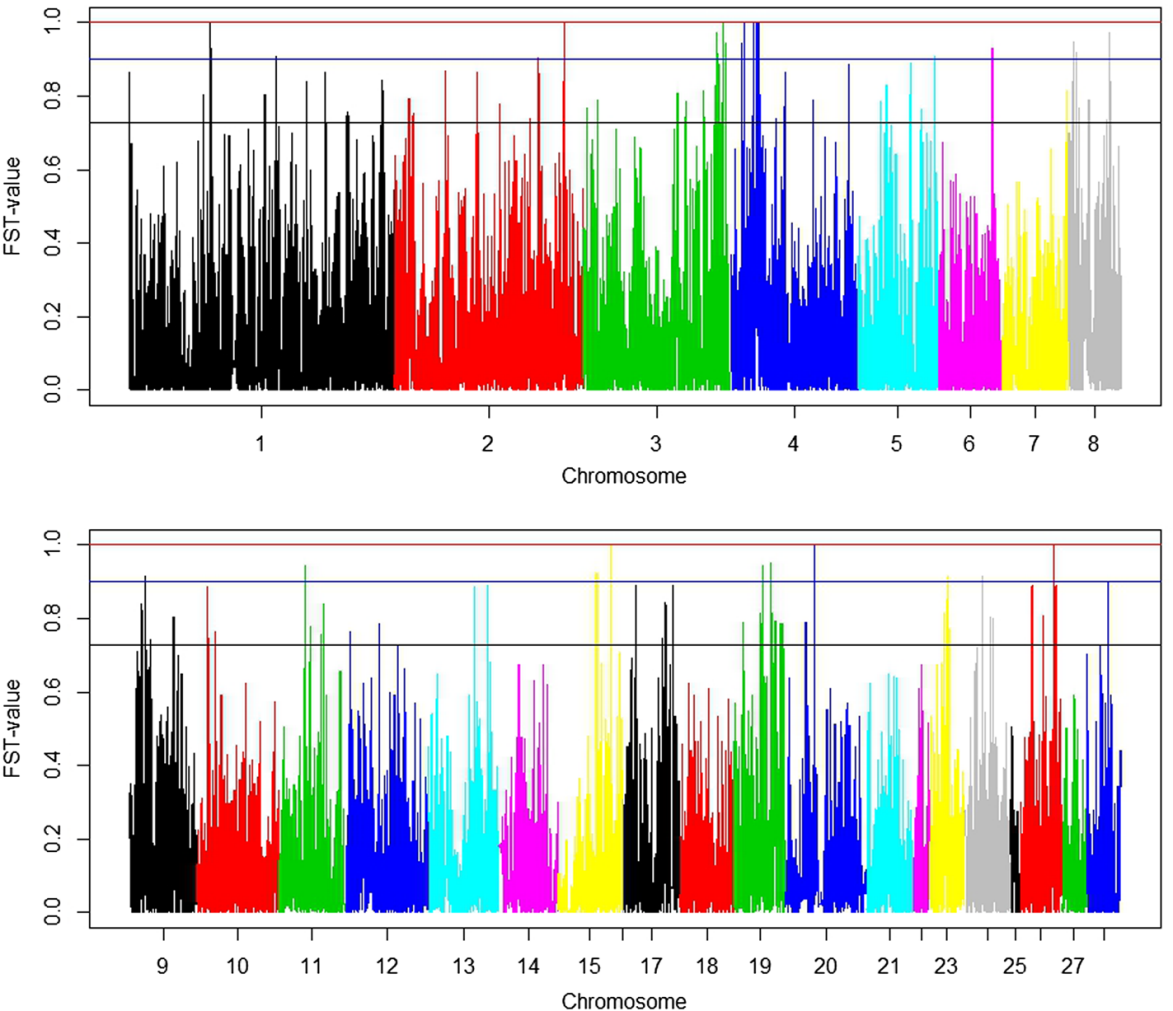
Manhattan plots of F_ST_-indexes. The *top panel* shows the F_ST_-values of each marker from chromosomes 1 to 8 and the *bottom panel* for chromosomes 9 to 28. The top threshold value indicates the genome-wide significance level p_genome wide_ <0.05; the middle and bottom threshold values are the nominal significance levels p_nominal_ ≤5 × 10^−5^ and p_nominal_ ≤5 × 10^−4^, respectively

**Fig. 3:**
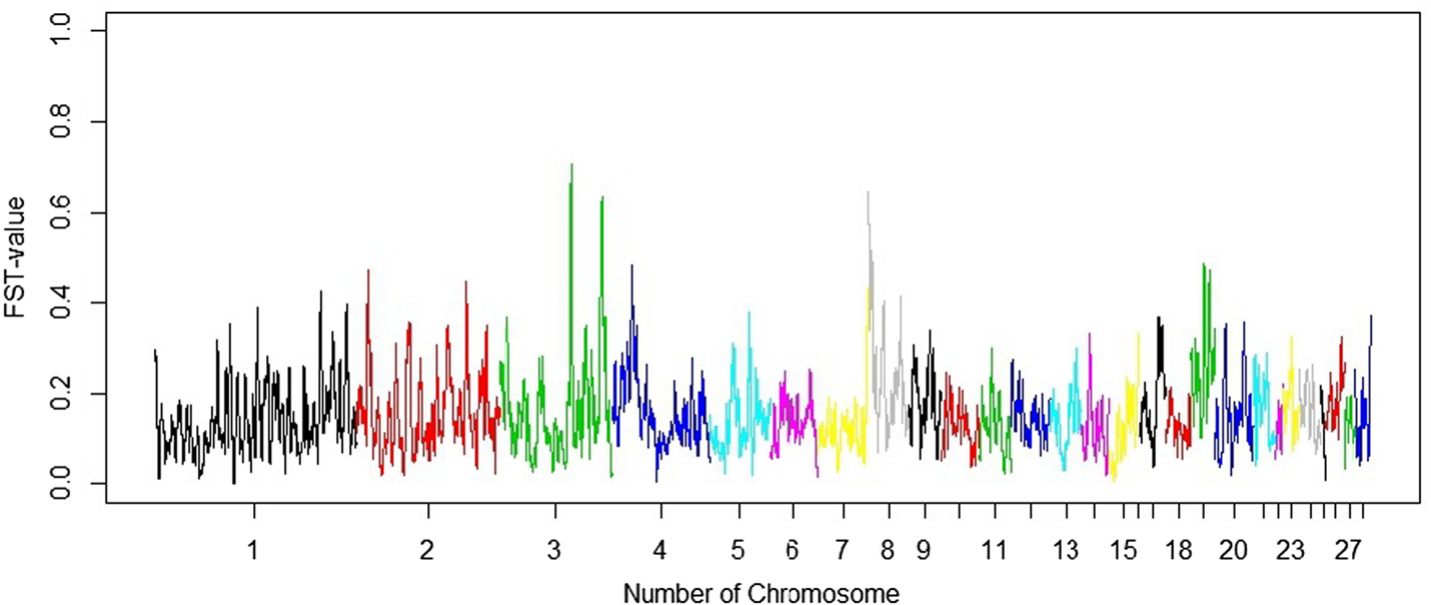
Manhattan plots of F_ST_-indexes in a sliding window of 25 consecutive SNPs

An overall F_ST_ index of 0.15 was estimated for the whole set of SNPs. The number of significant F_ST_ values is in Table 2. FDR for the significant SNPs were low, even at the relaxed significance level. The 17 genome-wide significant SNPs had an F_ST_ value of 1, i.e. alleles were divergently fixed in the two lines. A full list of significant SNPs is in Additional file 1: Table S1. Manhattan plots of the F_ST_ values are in Fig. 2. Most of the genome-wide significant SNPs are located on chromosome 4, followed by chromosome 3. The results of the sliding window approach (Fig. 3) revealed five distinct peaks, i.e. two on chromosome 3, and one on each of chromosomes 4, 8, and 19. Thirteen clusters with at least two significant SNPs were identified (Table 3). Based on Fig. 3, two clusters were observed on chromosomes 3, 4 and 19. These clusters harboured several genome-wide significant SNPs, especially the cluster on chromosome 4. The size of the clusters is small, except for those on chromosomes 3 and 4. Among the 17 genome-wide significant SNPs, 12 are located within the identified clusters.

**Table 2:** Number of significant SNPs, F_ST_-indexes and FDR of significant SNPs for three levels of significance

Significance level	Number of SNPs	F_ST_-index	FDR
p_genome_ _wide_			
<0.05	17	1.000	<0.001
p_nominal_			
≤5 × 10^−5^	49	0.901	≤0.015
p_nominal_			
≤5 × 10^−4^	276	0.730	≤0.034

**Table 3:** Number of clusters, chromosomes and chromosomal position in bp, length in Mb and number of significant SNPs (significant level p_nominal_ ≤5 × 10^−5^ and p_gemome wide_ ≤0.05) in each cluster

Cluster number	Chr.	Start/end position in bp	Length in Mb	Number of SNPs p_nominal_ ≤5 × 10^−5^	Number of SNPs p_gemone wide_ ≤0.05
1	1	58.108.441–58.537.760	0.43	4	1
2	3	103.609.224–105.597.337	1.40	5	0
3	3	108.252.363–109.945.836	1.69	2	1
4	4	10.364.490–10.575.112	0.21	3	3
5	4	18.580.845–21.323.065	2.74	7	7
7	6	31.974.670–32.086.164	0.11	2	0
8	8	4.002.499–4.211.591	0.21	2	0
9	8	23.892.743–23.911.149	0.02	2	0
10	11	11.015.338–11.139.271	0.12	2	0
11	15	7.826.821–7.879.094	0.05	2	0
12	19	5.204.468–5.273.813	0.07	2	0
13	19	6.883.105–6.896.487	0.01	2	0

## Discussion

One of the main reasons for establishing short-term selection experiments is to demonstrate that selection results in a selection response and thus, that it is feasible to breed for the trait under consideration. With regard to this, the selection experiment described in this study was moderately successful since selection response became immediately visible and the mean trait values of the two divergent selection lines differed for all generations, with the mean for line HFP always greater than that for line LFP (Fig. 1). Although not formally tested, it can be reasonably assumed that the consistent difference in the means of the number of FP bouts for both lines represents a true difference rather than a sampling effect, which was also stated by Kjaer et al. [1] and Su et al. [2]. This is also supported by the small standard errors estimated for the means (Fig. 1). The initial selection response in line HFP could not be maintained in subsequent generations. The reason is that it was often not possible to retain the animals with the highest estimated breeding value as parents to breed the next generation because of handling problems and increased mortality rates with these birds. This limited the selection intensity and hence genetic progress.

The data were analyzed with two very simple models, because no information was available on the observer, the pen, or other effects known to influence FP behaviour. The generation effect captured part of these effects. However, inclusion of this effect was a compromise since it probably captured a least part of the genetic progress. To some extent, the two models produced different results. A formal model comparison would be possible by assessing model predictive ability using cross-validation, but this was beyond the aim of our study. The dataset was too small for cross-validation, especially with the generation structure in the data.

**Fig. 4:**
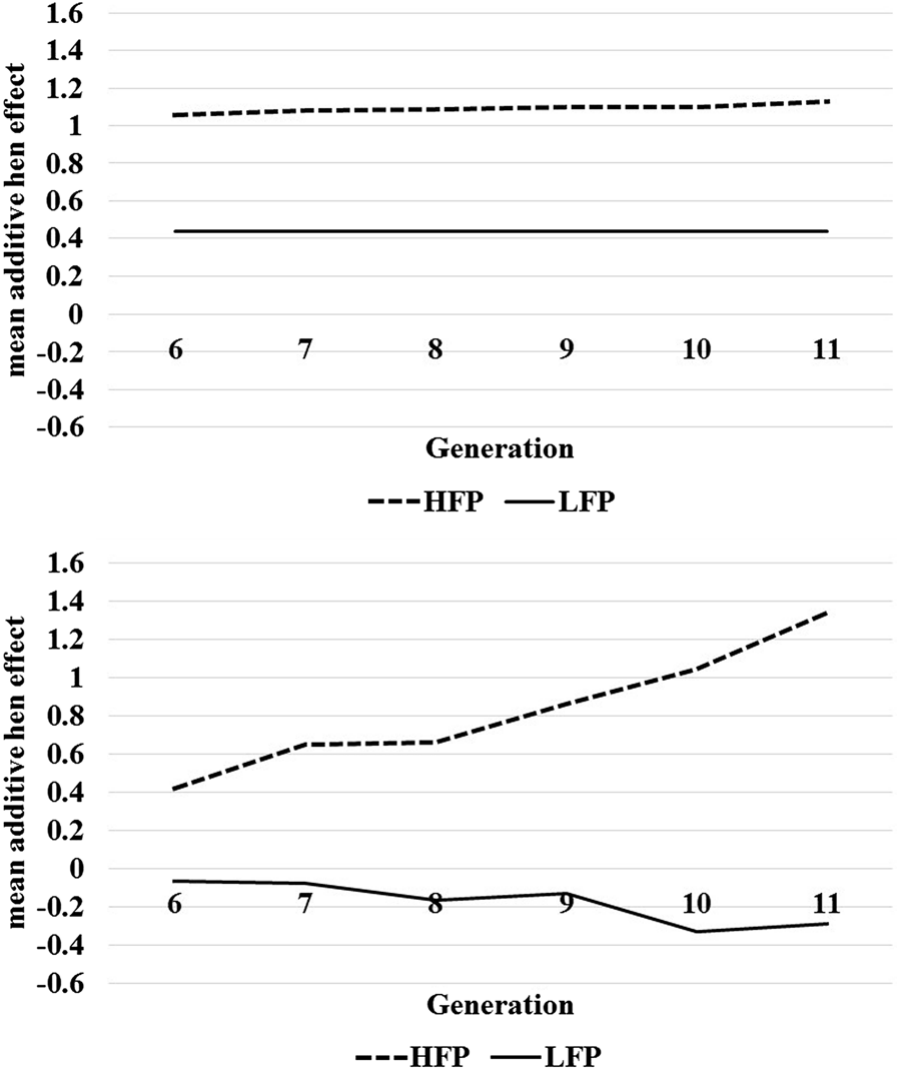
Selection response in the high (HFP) and low (LFP) feather pecking lines across generations. The mean additive hen effects were estimated with a linear mixed model (*top panel*) and with a Poisson model (*bottom panel*). The estimated intercept was added to the additive hen effects

It seems that the linear model attributed more variance to the generation effect, while in the Poisson model the additive genetic variance was greater (Table 1). In addition, the linear model estimated an additive genetic variance close to 0 in line LFP, while the Poisson model did not. Hence, although Fig. 1 suggests that line LFP is close to reaching a selection limit, the trend of the animal effects estimated with the Poisson model across generations still revealed a small selection response (Fig. 4). This response is not detected based on the trend of the mean additive effects estimated with the mixed linear model, as expected given the low additive genetic variance. The estimated heritability for line HFP was at the lower bound of the range of values reported in the literature (see “Background” section). For line HFP, the Poisson model revealed a continuous selection response (Fig. 4), which was not detectable with the mixed linear model. It seems that, in the mixed linear model, the generation effect completely captured the small genetic progress that was gained over generations, which was not the case in the Poisson model. The larger amount of variance explained by the generation in the mixed linear model compared to the Poisson model (Table 1) supports this explanation. The rank correlations between additive genetic effects of animals estimated with the mixed linear and Poisson models were equal to 0.74 (HFP line) and 0.68 (LFP line).

In analyses for the detection of selection sweeps, one of the main challenges is to separate drift and selection effects. In our study, due to the complete knowledge of the demographic history of the two selected lines since their common base population, we were able to model drift effect stochastically. This led to the detection of significant SNPs and selection signatures. The histogram of real and simulated (pure drift model) F_ST_ values is in Additional file 2: Figure S1. Compared to the histogram of simulated F_ST_, a thick tail is observed for the histogram of real F_ST_ values, which is likely due to the effect of selection. The applied test statistic is somewhat conservative, because the simulated gene frequency in the base population was set to 0.5, for which genetic drift is highest.

Based on the assumption that selection affects several consecutive SNPs, criteria to build a cluster of SNPs were defined, and based on these criteria, 13 clusters were identified. Most clusters were small and included only few significant SNPs (Table 3). The extent of LD for the SNPs included in this study for the two lines is not known. However, the drift that is operating during the selection experiment is expected to create a greater long-range LD within the lines compared to within the base population. In addition, selection results in LD around the functional gene. Given the relatively short selection period, the clusters that point to selection sweeps can be expected to be large. This might hold true for selection sweeps that are present only within one line, which, however, cannot be detected with the F_ST_ approach applied. Two interesting clusters were slightly larger than 2 Mb and included multiple significant SNPs on chromosomes 3 and 4 (Table 3). In addition, the 17 genome-wide significant SNPs were not randomly distributed across the genome, but, in most cases, located within the clusters, which supports the presence of selection sweeps around these clusters.

In contrast to quantitative genetic studies related to FP behaviour, to our knowledge, only a few QTL mapping experiments have been conducted and were mostly based on microsatellite linkage analysis (e.g. [13, 26, 27]). Buitenhuis et al. [26] reported a QTL for FP on chromosome 1, two on chromosome 2 and one on chromosome 10. We also identified clusters with selection sweeps on these chromosomes (Table 3), but a fine comparison of the QTL positions on these chromosomes is limited by the wide confidence intervals in QTL linkage studies. Biscarini et al. [28] performed an across-line SNP association study for genetic effects on feather damage in nine genetic lines and reported that the gene *HTR2C* (*5*-*hydroxytryptamine* (*serotonin*) *receptor 2C*, *G protein*-*coupled*) is associated with FP behaviour.

Molecular analyses suggested putative candidate genes for feather pecking behaviour [29–31]. According to Keeling et al. [29], the *PMEL 17* (*premelanosome protein*) gene affects plumage melanisation and the amount of feather pecking received. Two other candidate genes, *dopamine receptor D4* (*DRD4*) and *DEAF1 transcription factor* (*DEAF1*), have been shown to be associated with FP behaviour [30]. Gene expression analyses with brain tissues collected from individuals of the same HFP and LFP lines as those used here have led to the identification of six candidate genes, namely *HTR1B* (*5*-*hydroxytryptamine* (*serotonin*) *receptor 1B, G protein*-*coupled*), *SIP1* (*Smad interacting protein 1*), *PSEN1* (*presenilin*-*1*), *GLUL* (*glutamate*-*ammonia ligase*), *TSPO* (*translocator protein*) and *MAOA (monoamine oxidase A*), which may be involved in FP behaviour [31]. However, none of these candidate genes were located in the 13 cluster regions identified in our study.

## Conclusions

In conclusion, the use of a Poisson model is advantageous to analyze data on FP behaviour, because the assumptions made by the linear model are too heavily violated. This is especially the case if the number of FP bouts is small and the distribution is heavily peaked at 0, as is the case for line LFP.

The F_ST_-based approach that we applied to the genotypic data from individuals of the last generation of lines HFP and LFP was suitable to map selection signatures. Only a few individuals had to be genotyped and it was not necessary to perform individual phenotyping in addition to routine phenotyping. The non-random distribution of genome-wide significant SNPs indicates the presence of selection sweeps. A more detailed analysis of e.g. putative gene effects or the explained variance can only be done by using linkage or association mapping experiments. We have set up a large F2 design from lines HFP and LFP. The individuals of this experimental cross were phenotyped for a number of behaviour traits [10, 14] and are being genotyped using a high-density SNP chip. In the near future, we shall carry out QTL mapping using this F2 population and we shall combine the results with those obtained here as reported in Schwarzenbacher et al. [32] in order to detect and confirm QTL that affect FP behaviour.

## Additional files

10.1186/s12711-015-0154-0. SNPs with a genome-wide significance level p_genome wide_ < 0.05 and their position (bp) on the chromosome, F_ST_-value and cluster number based on Table 3 of the main text.
10.1186/s12711-015-0154-0 Histogram of real (top panel) and simulated (bottom panel) F_ST_-indexes. Figure S1 shows the histogram of the real and the simulated F_ST_-indexes.
