# Supplementary material for: Genetic parameters and signatures of selection in two divergent laying hen lines selected for feather pecking behaviour
Source: Genet Sel Evol. 2015 Sep 30;47:77. doi: 10.1186/s12711-015-0154-0 (PMC4589119; doi:10.1186/s12711-015-0154-0)
Supplement: Supplementary file 1 — 10.1186/s12711-015-0154-0. SNPs with a genome-wide significance level pgenome wide < 0.05 and their position (bp) on the chromosome, FST-value and cluster number based on Table 3 of the main text. [file 12711_2015_154_MOESM1_ESM.docx]

**Table S1 SNPs with a genome wide significance level p_genome wide_ < 0.05 and their position (bp) on chromosome, F_ST_-value and cluster number from Table 3 of the main text.**

| SNP | Chromosome | Position (bp) | F_ST_-value | Cluster from table 3 |
| --- | --- | --- | --- | --- |
| GGaluGA019372 | 01 | 58108441 | 1.00 | 1 |
| Gga_rs16139375 | 02 | 138867071 | 1.00 | - |
| GGaluGA238408 | 03 | 108252363 | 1.00 | 3 |
| Gga_rs13643399 | 04 | 10364490 | 1.00 | 4 |
| Gga_rs13643409 | 04 | 10387283 | 1.00 | 4 |
| GGaluGA245350 | 04 | 10575112 | 1.00 | 4 |
| GGaluGA248019 | 04 | 16462888 | 1.00 | - |
| Gga_rs16371032 | 04 | 18580845 | 1.00 | 5 |
| Gga_rs10725958 | 04 | 18594711 | 1.00 | 5 |
| Gga_rs15508371 | 04 | 18762763 | 1.00 | 5 |
| GGaluGA248971 | 04 | 21004836 | 1.00 | 5 |
| Gga_rs16371453 | 04 | 21226700 | 1.00 | 5 |
| Gga_rs14436318 | 04 | 21308984 | 1.00 | 5 |
| GGaluGA49012 | 04 | 21323065 | 1.00 | 5 |
| Gga_rs15024957 | 15 | 10460502 | 1.00 | - |
| Gga_rs10726111 | 20 | 5064598 | 1.00 | - |
| Gga_rs14300656 | 26 | 4035940 | 1.00 | - |
