# Supplementary figures and images for: Genetic parameters and signatures of selection in two divergent laying hen lines selected for feather pecking behaviour
Source: Genet Sel Evol. 2015 Sep 30;47:77. doi: 10.1186/s12711-015-0154-0 (PMC4589119; doi:10.1186/s12711-015-0154-0)

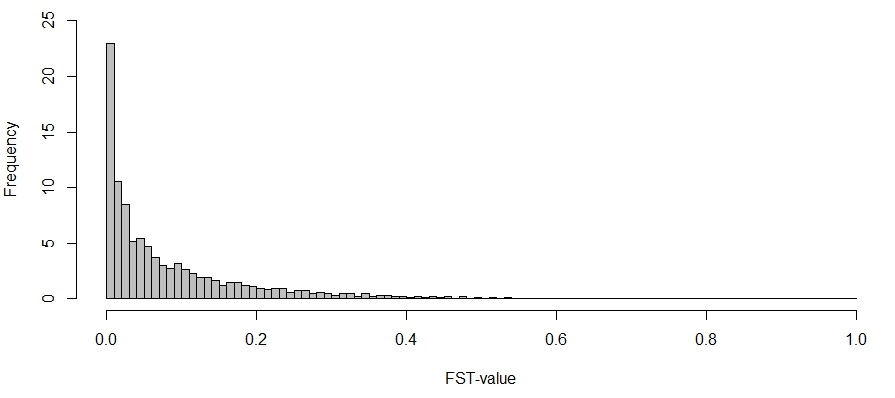

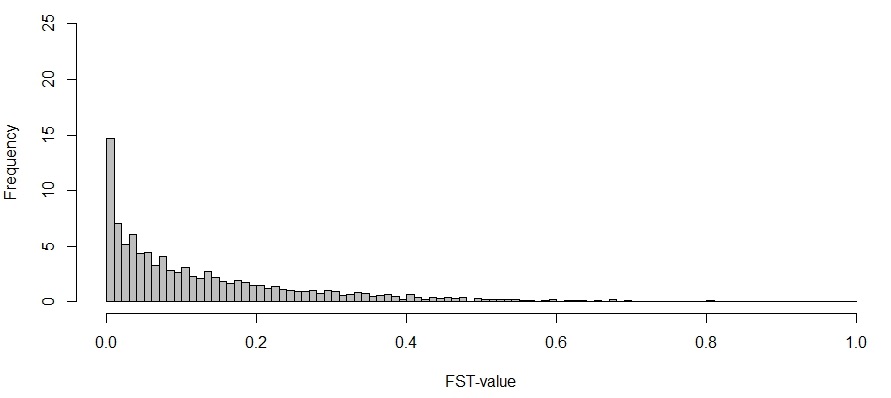

Supplement: Supplementary file 2 — 10.1186/s12711-015-0154-0 Histogram of real (top panel) and simulated (bottom panel) FST-indexes. Figure S1 shows the histogram of the real and the simulated FST-indexes. [file 12711_2015_154_MOESM2_ESM.docx]
